# ﻿Revision of the subgenus Mesopraocis Flores & Pizarro-Araya of the Neotropical genus *Praocis* Eschscholtz (Coleoptera, Tenebrionidae, Pimeliinae)

**DOI:** 10.3897/zookeys.1100.78769

**Published:** 2022-05-12

**Authors:** Gustavo E. Flores, Jaime Pizarro-Araya

**Affiliations:** 1 Instituto Argentino de Investigaciones de las Zonas Áridas (IADIZA), Laboratorio de Entomología, Centro Científico Tecnológico CONICET, Av. Ruiz Leal s/n, Parque General San Martín, 5500 Mendoza, Argentina Instituto Argentino de Investigaciones de las Zonas Áridas (IADIZA), Laboratorio de Entomología Mendoza Argentina; 2 Laboratorio de Entomología Ecológica (LEULS), Departamento de Biología, Facultad de Ciencias, Universidad de La Serena, Casilla 554, La Serena, Chile Universidad de La Serena La Serena Chile; 3 Grupo de Artrópodos, Sistema Integrado de Monitoreo y Evaluación de Ecosistemas Forestales Nativos (SIMEF), Chile Grupo de Artrópodos, Sistema Integrado de Monitoreo y Evaluación de Ecosistemas Forestales Nativos (SIMEF) La Serena Chile; 4 Instituto de Ecología y Biodiversidad (IEB), Santiago, Chile Instituto de Ecología y Biodiversidad (IEB) Santiago Chile

**Keywords:** Atacama, Chile, conical pegs sensilla, fossorial adaptations, *Mesopraocis*, new species

## Abstract

The second part of a revision of the species of the genus Praocis Eschscholtz is presented. The subgenus Mesopraocis Flores & Pizarro-Araya, distributed in northern Chile from 25° South to 31° South, is revised. This article includes a redescription of the subgenus, redescriptions of its species, an identification key, and a discussion on morphological adaptations of the species to fossorial life. Habitus photographs, scanning electron micrographs of head, antennae, prosternum, abdomen, and protibiae, illustrations of genital features, and distribution maps are presented. Conical pegs sensilla on tibiae are described and illustrated using SEM for the first time for a South American tenebrionid species. A new *Praocis* species is described: Praocis (Mesopraocis) arenicola sp. nov. Praocis (Mesopraocis) flava Kulzer, 1958 is synonymised with P. (M.) pilula Laporte, 1840. Other species of the subgenus are: P. (M.) calderana Kulzer and P. (M.) nitens Kulzer. A statement on the variation in the number of antennomeres in P. (M.) pilula is appended.

## ﻿Introduction

*Praocis* Eschscholtz is the type genus of the Praociini, an endemic Neotropical tribe of Pimeliinae with 149 species/subspecies arranged in 15 genera (Flores and Pizarro-Araya 2012; Flores and Giraldo 2020). *Praocis* is the most species-rich genus of the tribe (56% of the species) with 77 species and 8 subspecies arranged in nine subgenera (Flores and Pizarro-Araya 2014). Its species are distributed in arid and semiarid lands of western and southern South America from central and southern Peru, through central and southern Bolivia, to the southern part of Patagonia in Argentina and Chile (Flores and Pizarro-Araya 2014). A complete statement on the taxonomy of the genus, distribution, and subgeneric classification of *Praocis* is provided in Flores and Pizarro-Araya (2012, 2014).

Of the nine subgenera of *Praocis*, six of them inhabit northern and central Chile from 25° South to 42° South: *Praocis* s. str., *Orthogonoderes* Gay & Solier, *Filotarsus* Solier, *Mesopraocis* Flores & Pizarro-Araya, *Postpraocis* Flores & Pizarro-Araya, and *Anthrasomus* Guérin-Méneville (Flores and Pizarro-Araya 2014); of these, three are endemic to this area (*Praocis* s. str., *Mesopraocis* and *Anthrasomus*), while the remaining three are also present in Peru, Bolivia, and Argentina (Flores and Pizarro-Araya 2014). Taking into account this information, northern and central Chile is the area with the greatest sympatry of subgenera and major diversity of species for the genus (Peña 1966; Vidal and Guerrero 2007; Flores and Pizarro-Araya 2012, 2014). According to the biogeographic scheme of Morrone (2015), these species inhabit the biogeographic provinces Coquimban, Santiagan, Maule, and Valdivian Forest.

As with most members of Pimeliinae, species of *Praocis* are flightless (Matthews et al. 2010) and possess morphological, physiological, and ethological adaptations (Cloudsley-Thompson 2001) that have increased survival in arid environments and allow for more efficient exploitation of a great number of niches (Carrara and Flores 2015). Within northern and central Chile most *Praocis* species are distributed in the following vegetal formations of Gajardo (1994): Huasco coastal desert, plains florid desert, hills florid desert, coastal steppe scrub, forest steppe scrub, and arborescent steppe scrub (Flores and Pizarro-Araya 2012, 2014). In these habitats, species of subgenera *Filotarsus* and *Anthrasomus* were collected under stones in clayey soils, *Praocis* s. str., *Orthogonoderes*, and *Postpraocis* include psammophilous species found in well-consolidated sands with more or less abundant vegetation (Flores and Pizarro-Araya 2012, 2014), and *Mesopraocis* species are the only inhabitants of loose sands mostly in coastal grassy dunes with scattered vegetation.

Praocis (Mesopraocis) Flores & Pizarro-Araya, 2014 comprises four species endemic to Northern Chile, inhabiting coastal areas from 25° South (Paposo, Antofagasta Region) to 31° South (Caleta Limarí, Coquimbo Region). The objectives of this study are to revise the subgenus Praocis (Mesopraocis) by incorporating new characters from external morphology and genital features, to describe a new species, to detail their geographic distribution, and to analyze the fossorial adaptations of the species to sandy habitats. In Flores and Pizarro-Araya (2014) we presented a brief description of the subgenus based on detailed comparison with the other subgenera of *Praocis* and a key to the subgenera; in this paper we present a more detailed description of Praocis (Mesopraocis) for improved diagnostics and for future phylogenetic purposes.

## ﻿Materials and methods

The present study is based on examination of specimens borrowed from the following collections and curators (we follow Arnett et al. 1993 where possible for collections codens): American Museum of Natural History, New York, USA (**AMNH**, Lee Herman), Field Museum of Natural History, Chicago, USA (**FMNH**, Alfred Newton, Margaret Thayer), Hungarian Natural History Museum, Budapest, Hungary **HNHM** (Ottó Merkl), Instituto Argentino de Investigaciones de las Zonas Áridas, Mendoza, Argentina (**IADIZA**, Sergio Roig-Juñent), Laboratorio de Entomología Ecológica, Universidad de La Serena, Chile (**LEULS**, Jaime Pizarro-Araya), Museo Nacional de Historia Natural, Santiago, Chile (**MNNC**, Mario Elgueta Donoso), Natural History Museum, Basel , Switzerland (**NHMB**, Eva Sprecher), C.A. Triplehorn Insect Collection, The Ohio State University (**OSUC**, Charles A. Triplehorn), Universidad de Concepción, Concepción, Chile (**UCCC**, Juan Carlos Ortíz).

For this research we have searched for the type series of the species Praocis (Mesopraocis) pilula Laporte, 1840. According to Horn and Kahle (1935–1937) and Cambefort (2006) some type specimens of François Laporte Comte de Castelnau could be deposited in the Muséum National d’Histoire Naturelle, Paris, France (**MNHN**) but even with the help of Claude Girard and Antoine Mantilleri in MNHN, no type specimens of *Praocis* of this author were found there. A more recent research established that Laporte donated his entomological collection to the Smithsonian Institution (at that time National Museum of the United States) in 1841 (Evenhuis 2012) including many name-bearing types of his species described until 1840 and it is most probable that these were destroyed by the fire at Smithsonian Institution in the year 1865 (Evenhuis 2012).

Measurements were recorded with a micrometer eyepiece microscope. Body length was measured dorsally, along the midline, from the anterior margin of the labrum to the apex of elytra. Terminology used in descriptions follows recent papers dealing with the genus *Praocis* (Flores and Pizarro-Araya 2012, 2014) except “lateral expansion of frons” is replaced with epicanthus, “proepisternum” is replaced with hypomeron, “mesosternum” with mesoventrite, and “metasternum” with metaventrite (Matthews et al. 2010). Terminology of the foreleg was taken from Doyen (1984: Fig. 41). Dissection methods are those used by Tschinkel and Doyen (1980) for genital structures. Terminology of male genitalia was taken from Flores (1996); for basal lamina of tegmen/lateral styles length (B/E) and median lobe/tegmen length (L/T) we used the ratios proposed by Flores (1996). Terminology of female genitalia and the ratio paraproct/coxite length (P/C) are those proposed by Tschinkel and Doyen (1980) and Doyen (1994). Following the suggestion of Kamiński et al. (2020) to assess homologies in the morphology of female genitalia, we used characters from a recent study of another genus of Praociini, *Parapraocis* Flores & Giraldo (Flores and Giraldo 2020).

Terminology of protibial sensilla follows Crava et al. (2019) and for conical pegs is based on Matthews et al. (2010). In protibiae of Praocis (Mesopraocis) species two types of conical pegs (= conical processes of Medvedev 1965) are present. Conical peg type 1 (CP1), which is inserted on a large socket, presents a grooved cuticle that smoothens at the round tip; its cuticular shaft is straight and the length is twice the width (Fig. 2C). Conical peg type 2 (CP2) is also inserted on a large socket and with a less evident grooved cuticle that smoothens at the pointed tip; its cuticular shaft is straight as well and the length is about 4.5 times the width (Fig. 2D). Both types of conical pegs are similar in shape to the conical peg type 2 defined in the ovipositor tip of the fly *Drosophilasuzukii* (Matsumura) (Crava et al. 2019), and to the sensilla named tibial spines by Seada and Hamza (2018) around the distal end of protibiae of the tenebrionid *Triboliumcastaneum* (Herbst). Crava et al. (2019) performed serial transmission electron microscopy (TEM) at the base of conical peg type 2, showing the presence of a solid cuticular shaft, a small internal lumen without sensory neurons, and a single sensory neuron with a distal tubular body attached at the base of the peg (Crava et al. 2019: Fig. 5G–I) suggesting that these structures host mechanosensory neurons. Conical pegs in tibiae were also previously imaged for other tenebrionid genera such as *Planostibes* Gemminger & Harold (Opatrini) (Schimrosczyk and Iwan 2016) and *Trachyscelis* Latreille (Trachyscelini) (Nabozhenko and Purchart 2017).

The phylogenetic species concept of Wheeler and Platnick (2000) was employed to define a species as “the smallest aggregation of (sexual) populations or (asexual) lineages diagnosable by a unique combination of character states”. It was used in recent taxonomic works (Smith 2013; Smith and Wirth 2016; Kamiński et al. 2019). Species are diagnosed by the presence of autapomorphic morphological characters and/or a unique combination of homoplastic characters shared by all of the specimens assigned to a species (Smith and Wirth 2016).

**Figure 1.**
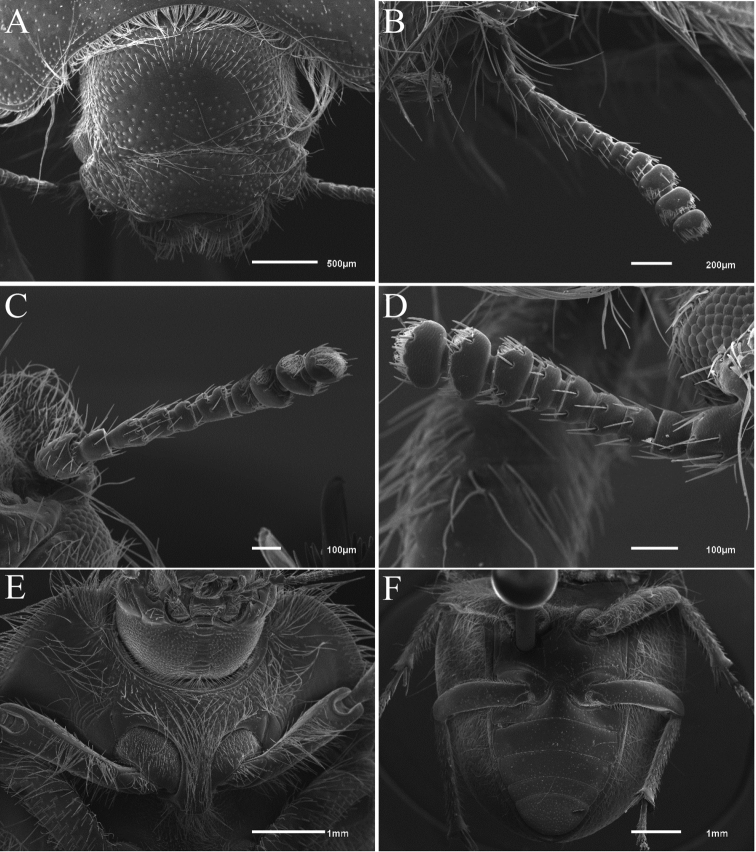
Scanning electron micrographs of body details of Praocis (Mesopraocis) pilula**A** head in dorsal view, 45× **B** antenna with 11 antennomeres, dorsal view, 70× **C** antenna with 10 antennomeres, ventral view, 100× **D** antenna with 9 antennomeres, dorsal view, 160× **E** prosternum and hypomeron, 25× **F** meso, metaventrite, abdomen, and pseudopleuron, 17×.

**Figure 2.**
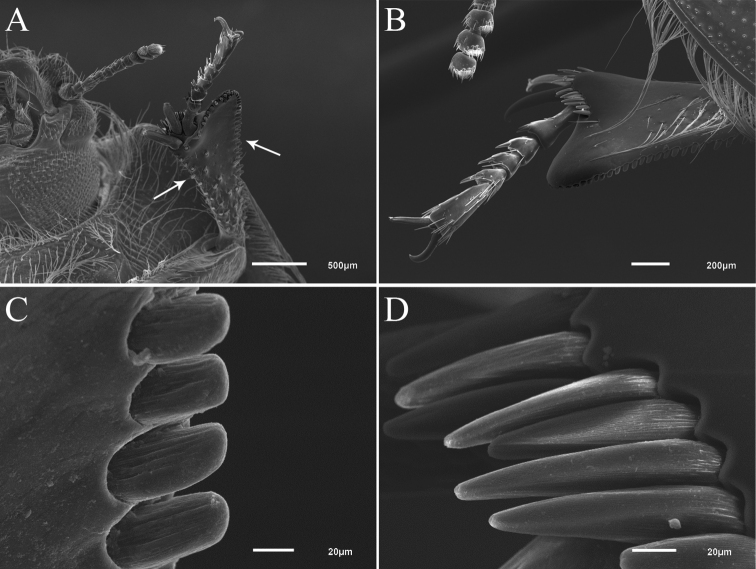
Scanning electron micrographs of body details of Praocis (Mesopraocis) pilula**A** protibia, posterior face, 37× **B** protibia, anterior face, 65× **C** conical pegs sensilla type 1 (CP1) (on outer margin of tibia), 700× **D** conical pegs sensilla type 2 (CP2) (on antero-distal margin of protibia), 750×. Arrows indicate conical pegs sensilla type 1.

Scanned electron micrographs (Figs 1A–F, 2A–D) were obtained using a JEOL JSM-6610 LV scanning electron microscopy. Digital images were taken with a Canon S50 adapted to a Leica MZ6 stereomicroscope. Final images (Fig. 4A–D) were merged with the image stacking freeware CombineZM (Hadley 2006). Drawings were made with a camera lucida adapted to a stereoscopic microscope.

Exact label data are cited only for the holotypes, where brackets delimit the text of individual labels and new lines on the same label are separated by a diagonal slash (/). Geographic coordinates of the collecting sites were recorded using a GPS Garmin eTrex, Vista C. The distribution map was generated using ArcMap 9.0 (Environmental Systems Research Institute, Redlands, California). For distribution of the species we used the biogeographic classification of Morrone (2015).

## ﻿Taxonomy

### ﻿Subgenus Praocis (Mesopraocis)

Taxon classificationAnimaliaColeopteraTenebrionidae

Flores & Pizarro-Araya, 2014

F3F16865-F1B8-571C-9D15-A62D4976CFDB

Praocis (Mesopraocis) Flores & Pizarro-Araya, 2014: 60. Type species: Praociscalderana Kulzer, 1958 (original designation).

#### Redescription.

Length 5.00–10.62 mm; habitus oval, convex; dorsal surface with short setae, ventral surface densely setose; pseudopleuron with long golden setae on upper surface, forming with the setae of the hypomeron a continuous row of long golden setae surrounding the body. Body, antennae, legs black, dark brown to light brown.

***Head*.** Clypeal anterior margin concave, extended beyond epicanthus; frontoclypeal suture distinct, as a vertical groove, not covered by frons, clypeus and frons at same level; clypeus and frons with round punctures, epicanthus subquadrate (Fig. 1A). Antennae equal in length in both sexes; antennomere 10 wider than long; apical tomentose sensory patches on antennomeres 9 and 10 in two areas subequal in size, on antennomere 11 on distal third (Fig. 1B–D).

***Thorax*.** Pronotum strongly convex, widest behind midpoint or at base, lacking carinae or striae; anterior margin concave, lacking carinate edge, width of anterior margin exceeding half the width of posterior margin; with lateral margins concave in anterior half and subparallel in posterior half (Fig. 4A–D); disc with sparse round punctures, each bearing a short, decumbent seta, visible at higher magnification (50×); punctures of disc smaller than punctures of elytron; prosternum horizontal, with carinate edge on anterior margin, broadened below gula (Fig. 1E); prosternal process subrectangular forming a straight angle, produced backwards, not reaching the midpoint of the space between pro, mesocoxae. Mesoventrite inclined forward, separated from prosternum (Fig. 1F). Hypomeron with shallow grooves not reaching lateral margin of pronotum, with a fringe of short or long golden setae below lateral margin of pronotum (Fig. 1E). Hypomeron with tubercles; mesepisternum and metepisternum with punctures. Metaventrite smooth on central area, with punctures on lateral thirds, and with two transverse grooves parallel to metacoxae (Fig. 1F). Metacoxal cavity closed laterally by metaventrite and abdominal ventrite 1.

***Elytron*** convex, surface punctate, lacking carinae or striae, lateral margin not defined; pseudopleuron with a fringe of long golden setae on anterior half or entire upper surface (Fig. 1F), forming with the setae of the hypomeron a continuous fringe of long golden setae surrounding the body; epipleuron distinct, finely ridged throughout (Fig. 1F), gradually widening anteriorly, anterior quarter four times as wide as posterior half, anterior margin reaching elytral humeri and posterior angle of pronotum. Wingless, subelytral cavity sealed.

***Legs*.** Distance between meso- metacoxae exceeding half mesocoxal length (Fig. 1F). Pro, mesofemora straight, metafemora curved inward. Femora with long, fine setae on anterior, posterior surfaces and dorsal fringe, abundant on pro, mesofemora, sparse on metafemora; ventral surface of profemora with a row of long setae on anterior edge. Protibiae explanate, apical process concave from behind (Fig. 2A), inner margin armed with a row of stout setae, outer margin concave; postero-distal, outer margins and posterior face with conical pegs sensilla type 1 (CP1) (Fig. 2A, B), antero-distal margin with conical pegs type 2 (CP2) (Fig. 2B), anterior and posterior faces (Fig. 2A, B) with long, fine setae (sensilla trichoidea) arising on punctures; posterior face of protibiae and inner, outer faces of meso, metatibiae with short, stout setae, arising on tubercles; meso, metatibiae with conical pegs type 1 on posterior face and type 2 on distal end. Pro, mesofemora longer than pro, mesotibiae; metafemora shorter than metatibiae; metatibiae straight. Tarsi bearing sparse decumbent setae on ventral surface; protarsomere 1 equal to combined length of tarsomeres 2–4, subequal to tarsomere 5 (Fig. 2B).

***Abdomen*.** Ventrites 1–4 with sparse punctures each bearing a short seta; ventrite 5 with sparse punctures on central area separated by two to four puncture diameters, on lateral thirds separated by one to two puncture diameters. Male sternites VII and VIII emarginated.

**Figure 3.**
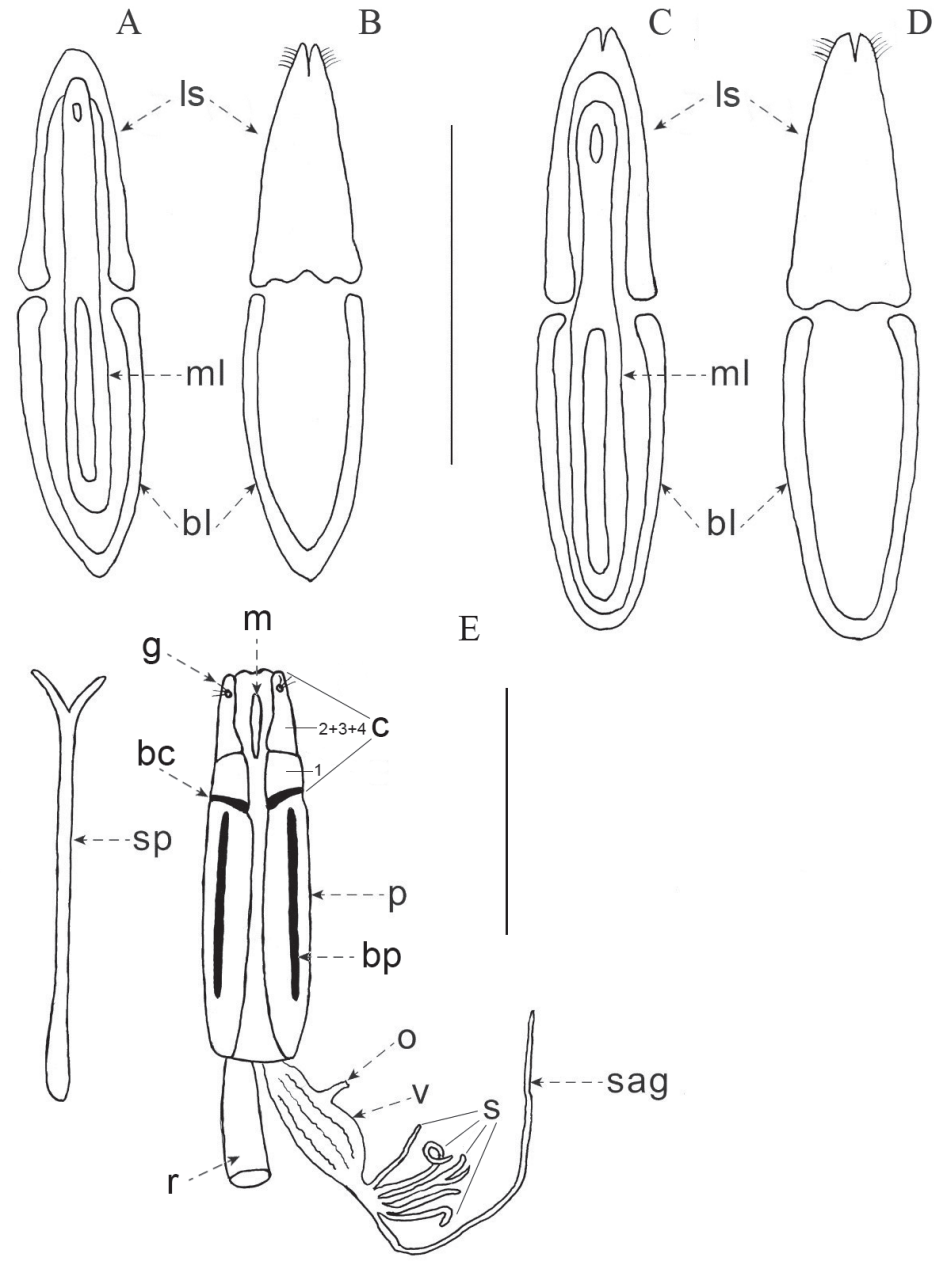
Male and female genitalia of Praocis (Mesopraocis) species **A-D** male **A, B**Praocis (Mesopraocis) calderana, dorsal and ventral views **C, D**Praocis (Mesopraocis) nitens, dorsal and ventral views. Abbreviations: bl, basal lamina of tegmen, ls, lateral styles of tegmen, ml, median lobe **E** ovipositor (ventral view), spiculum ventrale and internal female reproductive tract of Praocis (Mesopraocis) pilula. Abbreviations: bc, baculi of coxite, bp, baculi of paraproct, c, coxite, g, gonostyli, m, midventral sclerite, o, oviduct, p, paraproct, r, rectum, s, spermatheca, sag, spermathecal accessory gland, sp, spiculum ventrale, v, vagina, 1, 2, 3, 4, coxite lobes. Scale bars: 1 mm.

***Male terminalia and genitalia* (Fig. 3A–D).** Rods of spiculum gastrale V-shaped, joined at the apex. Dorsal membrane of proctiger concave, without sclerotized areas. Basal lamina of tegmen long (B/E > 1.0) (Fig. 3A, C). Lateral styles of tegmen distally close, with apex narrow, with setae on lateral margins (Fig. 3B, D), widest at base, and not overlapping median lobe dorsally. Median lobe tubulous, moderate (0.75 < L/T ≤ 1.00) and apex rounded (Fig. 3A, C).

***Female terminalia and genitalia* (Fig. 3E).** Spiculum ventrale with arms short, “V”-shaped. Paraprocts long (2.0 < P/C ≤ 3.0), with setae; proctigeral baculus equal to length of paraproct baculus; apicodorsal lobe of proctiger extending about ½ length of coxite. Coxites with setae, divided into two visible lobes: the basal lobe bears oblique baculi and the apical lobe is composed of the fully fused second, third and fourth lobes, which bears lateral gonostyli, basal lobe of coxite not extended over paraproct, separated from the apical lobe by a transverse pleat and shorter than the apical lobe; midventral sclerite equal width throughout. Vagina saccate. Spermathecal accessory gland longer than vagina, with duct not annulate. Spermatheca with six basal tubes or less, all similar in length and branching pattern.

#### Geographic distribution.

Species of Praocis (Mesopraocis) are endemic to northern and central Chile and occur from 25° South (Paposo, Antofagasta Region) to 31° South (Caleta Limarí, Coquimbo Region) in the Atacama and Coquimban biogeographic provinces (Morrone 2015) (Figs 5, 6). Up to two sympatric species have been recorded together in the distribution area of the subgenus. But all four species can be sympatric with the remaining species (Figs 5, 6) and there are no geographic barriers that separate species.

#### Habitat.

The distribution range of the subgenus extends from sea level to an altitude of ~1325 m. All Praocis (Mesopraocis) species are associated with coastal dunes stabilized with scattered vegetation and inland aeolian dunes located in the central valley (JPA pers. obs.) in the transitional coastal desert of Chile (Cortés-Contreras et al. 2013; Flores and Pizarro-Araya 2014). Some species have been recorded in Pacific islands such as Choros, Damas, and Gaviota from the Choros Archipelago (Alfaro et al. 2009). They are associated with shrubby and herbaceous vegetation (perennial and annual) characteristic of coastal dunes such as *Nolana* spp. (Solanaceae), *Rhodophiala* spp. (Amaryllidaceae), *Leucocoryne* spp. (Leucocoryneae), *Cristaria* spp. (Malvaceae).

#### Biology.

Adults have nocturnal habits, remaining buried in the sand during the day and appearing a short while after sunset (JPA pers. obs.), when sand surface cools down and night-moisture appears (Koch 1961). Adults eat flowers and detritus of dune vegetation and larvae feed on tubers and roots of dune plants (JPA pers. obs.). Laboratory observations on oviposition: the females dig a depth ranging from 10 to 20 cm in the substrate and laid eggs individually or in groups of 3 to 5. The egg chorion is covered by a protective layer of mucilage to which sand grains adhere during oviposition. The resulting sand layer is thought to act as both a thermal insulator against the wide temperature oscillations that daily occur in these semiarid environments and as a mimicry strategy against edaphic predators, such as carabid larvae (e.g., *Calosomavagans* Dejean), scorpions (e.g., *Brachistosternus* spp.), solpugids (e.g., *Ammotrechelis* spp., *Mummucia* spp.), and spiders (e.g., *Lycinus* spp.) (JPA pers. obs.).

#### Ecology.

In a taxonomic diversity study of epigean tenebrionids in the Choros Archipelago (Coquimbo Region), Praocis (Mesopraocis) pilula reached 5.9% of total abundance, with specimens recorded in coastal dunes of Choros, Damas, and Gaviota islands (Alfaro et al. 2009). In other research conducted in Coastal Cordillera near Punta de Choros (Coquimbo Region), specimens of Praocis (Mesopraocis) pilula were caught in paleodunes stabilized with vegetation, making up 3.1% of the total catch (Cortés-Contreras et al. 2013). Finally, Pizarro-Araya et al. (2012) performed a survey in continental dunes of Atacama Region and captured specimens of Praocis (Mesopraocis) calderana Kulzer, 1958 which were 2.8% of the total assemblage of terrestrial arthropods.

### ﻿Key to species of the subgenus Praocis (Mesopraocis)

**Table:** 

1	Pronotum with posterior angles not produced, central area of posterior margin extending beyond lateral extents of posterior margin; body widest behind elytral humeri; antennae reaching anterior quarter of lateral margin of pronotum; pseudopleuron with punctures equal density on all surface, separated by two to four puncture diameters from which emerge decumbent setae (Fig. 4A)	**Praocis (Mesopraocis) pilula Laporte**
–	Pronotum with posterior angles produced, central area of posterior margin not extending beyond lateral extents of posterior margin; body widest at prothorax; antennae reaching anterior third of lateral margin of pronotum; pseudopleuron with punctures separated by three to six puncture diameters on lower surface and separated by one to two puncture diameters on upper surface from which emerge erect setae	**2**
2	Upper margin of eyes lacking supraocular groove; antennomere 9 wider than long and equal length to 10; first metatarsomere twice as long as metatarsomeres 2–3 combined (Fig. 4B)	**Praocis (Mesopraocis) calderana Kulzer**
–	Eyes with supraocular groove, starting in the middle of upper margin of eye and ending on upper postgenal margin; antennomere 9 longer than wide or equal length than wide, and longer than 10; first metatarsomere 1.4–1.6 × longer than metatarsomeres 2–3 combined	**3**
3	Frons, epicanthus and elytra with impressed punctures, frons and epicanthus with puncture depth the same size than diameter, elytra with puncture depth larger than diameter; antennomere 9 longer than 11 and narrow than 10, antennomere 11 wider than long; posterior ¾ of epipleuron with a row of punctures the same diameter and depth than punctures on pseudopleuron (Fig. 4C)	**Praocis (Mesopraocis) nitens Kulzer**
–	Frons, epicanthus and elytra with shallow punctures, frons and epicanthus with puncture depth smaller than diameter, elytra with puncture depth the same size than diameter; antennomere 9 of equal length than 11 and of equal width to 10, antennomere 11 longer than wide; posterior ¾ of epipleuron with a row of punctures smaller diameter and depth than punctures on pseudopleuron (Fig. 4D)	**Praocis (Mesopraocis) arenicola sp. nov.**

### ﻿ Praocis (Mesopraocis) pilula

Taxon classificationAnimaliaColeopteraTenebrionidae

Laporte, 1840

0E1EDF56-E092-507E-B8E3-C4CC4D33DC08

**Figure 4.**
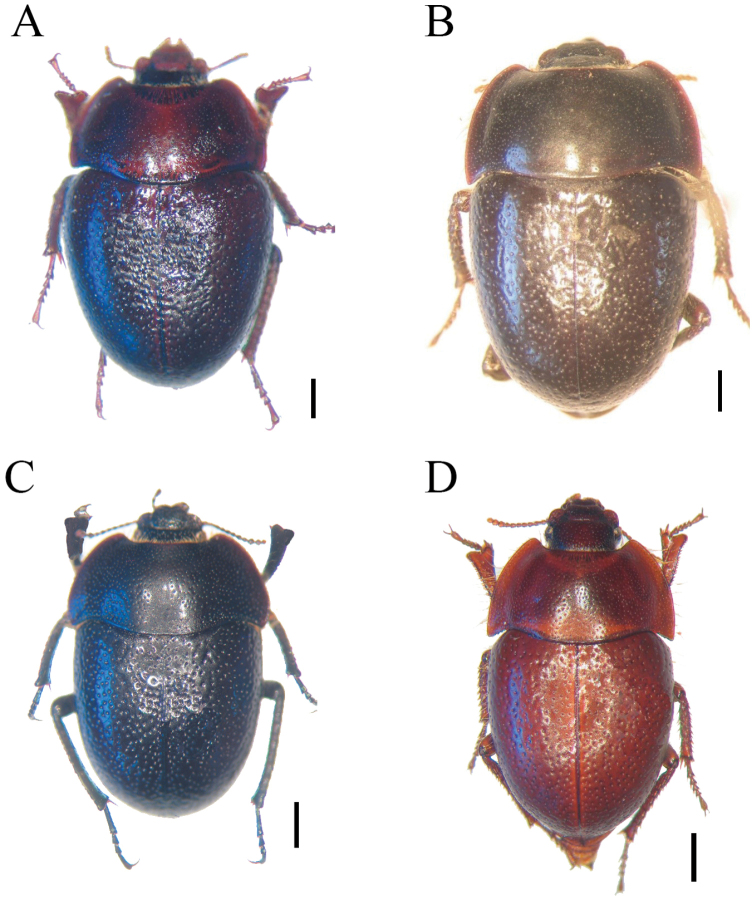
Habitus in dorsal view **A**Praocis (Mesopraocis) pilula**B**Praocis (Mesopraocis) calderana**C**Praocis (Mesopraocis) nitens**D**Praocis (Mesopraocis) arenicola sp. nov. Scale bars: 1 mm.

Figs 1A–F , 2A–D , 3E , 4A , 6

Praocis pilula Laporte, 1840: 187; Lacordaire 1859: 214; Gemminger and Harold 1870: 1905 (cat.); Philippi 1887: 732 (cat.); Gebien 1910: 261 (cat.); Gebien 1938: 400 (cat.); Blackwelder 1945: 523 (cat.); Kulzer 1958: 31 (rev.); Peña 1966: 431 (cat.); Vidal and Guerrero 2007: 73, 219; Pizarro-Araya et al. 2008: 273 (list.); Alfaro et al. 2009: 126 (ecol.); Cortés-Contreras et al. 2013: 98, 99 (ecol.); Flores and Pizarro-Araya 2014: 60 (list).
Coelus hirticollis Solier, 1840: 212; Solier 1851: 185 (rev.); Lacordaire 1859: 214. Synonymy by Lacordaire 1859: 214.
Praocis flava Kulzer, 1958: 32 (rev.); Peña 1966: 431 (cat.); Vidal and Guerrero 2007: 73; Pizarro-Araya et al. 2008: 273 (list.); Alfaro et al. 2009: 126 (ecol.); Flores and Pizarro-Araya 2014: 60 (list). New synonymy.

#### Redescription.

Length 5.00–9.25 mm. Body, antennae, legs black, dark brown to light brown, body widest behind elytral humeri. Head. Clypeus with punctures bearing short setae on centre and long setae at sides, width of anterior margin exceeding half the interocular width; frons with punctures bearing short setae on centre and long setae at sides and posterior half (Fig. 1A), border of punctures not elevated; frontoclypeal suture with short or long setae; eyes lacking supraocular groove; antennae reaching anterior quarter of lateral margin of pronotum; antennomeres 9 and 11 wider than long (Fig. 1B–C).

***Thorax*.** Pronotum with anterior angles rounded, not produced, remote from eyes and epicanthus, lateral margins declivous, disc convex reaching lateral margins (Fig. 4A); posterior margin convex, central area extending beyond posterior angles (Fig. 4A), posterior angles right with apex rounded, not produced. Hypomeron with a fringe of long golden setae below lateral margin of pronotum (Fig. 1E). Metaventrite with punctures on lateral thirds separated by one to two puncture diameters.

***Elytron*.** Pseudopleuron with abundant punctures from which emerge long, golden, decumbent setae, punctures equally dense on all surface, separated by two to four puncture diameters (Fig. 1F); epipleuron with a fringe of long golden setae along the edge, texture different than that of elytron, shiny, almost smooth, with sparse punctures and setae.

***Legs*.** Ventral surface of protrochanters with hair brush. Apical process of distal margin of protibiae equal to length of protarsomeres 1–3 combined; protibial length twice the width of distal margin. First metatarsomere 1.4–1.6 times longer than metatarsomeres 2–3 combined (Fig. 4A).

***Abdomen*.** Ventrites 1–3 with sparse punctures separated by two to four puncture diameters; ventrite 4 with sparse punctures separated by one to two puncture diameters; ventrite 5 with punctures on central area separated by two to four puncture diameters, on lateral thirds separated by one to two puncture diameters.

***Male genitalia*.** Basal lamina of tegmen equal width throughout, with base concave. Lateral styles of tegmen with proximal margin straight, slightly bisinuate at sides, with setae on distal 1/5 of ventral surface. Median lobe proximally narrow, half the width of tegmen, with apical aperture small.

***Intraspecific variation*.** Antennae can exhibit 9, 10 or 11 antennomeres, the same individual can bear both antennae with different number of antennomeres (Fig. 1B–D). If it has 11 or 10 antennomeres due to the fusion or lack of intermediate segments, antennomere 9 is equal length to 10 and 11 and antennomere 9 is equal width to 10 and wider than 11 (Fig. 1B–C); if the antenna has 9 antennomeres due to the fusion or lack of intermediate segments, antennomere 9 is equal length to 10 but shorter than 11 and antennomere 9 is equal width to 10 and 11 (Fig. 1D).

#### Notes on synonymy.

Kulzer (1958) described *Praocisflava* based upon six specimens that were probably tenerals (he stated light yellow colour), and pointed out that the antenna is shorter than the width of frons to separate this species from the other two known of the subgenus at this time: *P.pilula* and *P.calderana*. We examined the allotype of *P.flava* (NHMB) which has 11 antennomeres on the right antenna and 10 antennomeres on the left antenna. We studied hundreds of specimens of *P.pilula* and observed that antennae can bear 9, 10 or 11 antennomeres (Fig. 1B–D), with variation in the number of antennomeres in the same individual (right/left): 11/11, 11/10, 10/11, 10/10, 10/9, 9/10, 9/9 and this variation makes the antenna shorter than or equal to the width of frons. We conclude that *Praocisflava* is a synonym with *P.pilula* based on the characters shared by both nominal species stated in the identification key.

#### Type specimens.

Allotype of Praocis (Mesopraocis) flava: [Coquimbo] [Alotypus/ *Praocis*/ *flava* m./ det. H. Kulzer 1957] (NHMB).

#### Other material examined.

Chile. Atacama Region: Copiapo Province: E. Rodillo sand dunes 476 m, 27°1'5.3"S, 70°40'36."W, 30.x.2012, M. Elgueta, 9 (IADIZA), Rodillo sand dunes 14 m, 27°00'6.1"S, 70°47.4'10."W, 30.x.2012, M. Elgueta, 1 (IADIZA), Punta Frodden, 20 km N. Caldera, 26.iv.1956, L.E. Peña, 30 (FMNH), S. Caldera, 23-VI-1968, L.E. Peña, 5 (FMNH), Caldera, 30-XI-1980, L.E. Peña, 1 (FMNH), 4.x.1981, M. Elgueta, 1 (MNNC), Boca Río Copiapó, 13.vi.1968, L.E. Peña, 4 (FMNH), 70 km S. Copiapó, 27°22'S, 70°20'W, 18.viii.1966, E.I. Schlinger, M.E. Irwin, dune assoc, 4 (AMNH), E. Cerro Negro 1296 m, 27°7'8.6"S, 70°14.4'43."W, 30.x.2012, M. Elgueta, 10 (IADIZA), Puerto Viejo, 10.x.1980, L.E. Peña, 24 (FMNH), ix.1982, L.E. Peña, 3 (FMNH), Bahía Salada 2 m, 27°38'24.3"S, 70°54'38."W, 29.xi.2012, M. Elgueta, 1 (IADIZA), N Caleta Angosta sand dunes 11 m, 28°11'48"S, 71°09'22."W, 29.x.2012, M. Elgueta, 3 (IADIZA), Travesía, 20.viii.1978, Leg. J. Solervicens, 2 (MNNC), Punta Cachos, 01–04.xi.2012, J. Pizarro-Araya, 1 (LEULS). Huasco Province: Quebrada Carrizalillo, N. Huasco, 12.x.1980, L.E. Peña, 24 (FMNH), Cuesta Carrizalillo, 30.x.1980, L.E. Peña, 9 (FMNH), Carrizal Bajo, 8.xi.1965, L.E. Peña, 1 (FMNH), between Carrizal y Los Pozos, 8 m, 28°7'38"S, 71°9'53."W, 23.i.2010, E. Ruiz-Manzanos, F.M. Alfaro, J. Mondaca, 3 (LEULS), 20 (IADIZA), Los Pozos, 19.viii.2008, F.M. Alfaro, 6 (LEULS), Agua de Luna, 18.viii.2009, F.M. Alfaro, 2 (LEULS), 01–04.ii.2007, F.M. Alfaro, 12 (LEULS), Huasco bajo, 8.viii.1976, 2 (UCCC), Huasco, viii.1953, L.E. Peña, 16 (MNNC), viii.1957, L.E. Peña, 2 (FMNH), 22.x.1957, L.E. Peña, 4 (FMNH), 23.x.1980, L.E. Peña, 29 (FMNH), Huasco beach sand dunes 5 m, 28°27'24.5"S, 71°12'27."W, 28.x.2012, M. Elgueta, 1 (MNNC), 5 (IADIZA), 29.ix.1981, M. Elgueta, 4 (MNNC), Playa Tontando, 22.xi.2008, F.M. Alfaro, 4 (LEULS), Chañaral de Aceituno, 14.vi.1968, L.E. Peña, 11 (FMNH). Coquimbo Region: Elqui Province: Isla Gaviota, Los Choros, J. Pizarro-Araya, 1 (IADIZA), 8 (LEULS), Isla Damas, Reserva Nacional Pingüino de Humboldt, Los Choros, 3–6.viii.2006, D. Valdivia & F.M. Alfaro, 1 (IADIZA), 4 (LEULS), 3–6.viii.2006, P. Agusto & F.M. Alfaro, 7 (LEULS), 22–25.ix.2006, P. Gachón & C. Romero, 3 (LEULS), Punta de Choros, 3.vi.2005, D. Valdivia, 1 (IADIZA), 4.vi.2005, D. Valdivia, 1 (IADIZA), 11 (LEULS), 26.viii.2005, P. Agusto, 10 (LEULS), 27.viii.2005, R. Villalón, 13 (LEULS), 25.viii.2005, R. Villalón, 3 (LEULS), 21.xi.2005, J. Pizarro-Araya, 8 (LEULS), i.2007, J. Pizarro-Araya & F.M. Alfaro, 2 (LEULS), 31.x.2011, J. Pizarro-Araya & F.M. Alfaro, 4 (LEULS), Choros Bajos (NW El Tofo), 31.x.1961, L.E. Peña, 2 (FMNH), 15.vi.1968, L.E. Peña, 24 (FMNH), El Apolillado, Los Choros, 04.xi.2014, 2 (LEULS), Chungungo, Playa Blanca, 27.ii.2005, J. Pizarro-Araya, 6 (LEULS), Quebrada Porotitos, 16.ix.2005, R, Villalón, 1 (LEULS), Los Hornos, 29.58825°S, 71.29457°W, iii.2017, A. Zúñiga, M. Bläser, R. Predel, L. Ragionieri, 6 (IADIZA), Punta Teatinos, 7.i.1966, L.E. Peña, 31 (FMNH), 24.x.1992, P. Plandiura, 1 (IADIZA), 1 (LEULS), Caleta San Pedro, sand dunes 5 m, 29°52'54.3"S, 71°16'26."W, 7.xii.2012, M. Elgueta, 4 (IADIZA), Coquimbo, La Serena, ix.1947, L.E. Peña, 6 (MNNC), 1 (IADIZA), 28–31.viii.1947, L.E. Peña, 16 (MNNC), 10 (FMNH), 18 (IADIZA), 13.x.1957, L.E. Peña, 1 (FMNH), Coquimbo, 13.xi.1964, L.E. Peña, 7 (FMNH), 16.iii.1971, L. Álvarez, 1 (FMNH), 20.xii.1967, Valencia Leg., 21 (LEULS), La Herradura, 2.i.1969, L. Álvarez, 34 (FMNH), Guayacán, 20.ix.1969, G. Monsalve, 30 (FMNH), Totoralillo, 2–3.ix.1947, L.E. Peña, 13 (MNNC), 19 (FMNH), Totoralillo sand dunes, 11 m, 29°29'21.1"S, 71°19'2."W, 27.x.2012, M. Elgueta, 5 (IADIZA), Lagunillas, 4.ix.1947, L.E. Peña, 10 (MNNC), 13 (IADIZA), Morrillos, sand dunes 41 m, 30°8'56.4"S, 71°22'13."W, 27.x.2012, M. Elgueta, 1 (MNNC), 15 (IADIZA), Guanaqueros, 21.v. 1955, 3 (MNNC), 24.xi.1967, L.E. Peña, 18 (FMNH), N. Guanaqueros, sand dunes 4 m, 30°11’28"S, 71°24'22."W, 24.x.2010, M. Elgueta, 1 (MNNC), Tongoy, 28.x.1961, L.E. Peña, 9 (FMNH), 10.iii. 1967, L.E. Peña, 26 (FMNH), N. Tongoy sand dunes, 24 m, 30°15'15.5"S, 71°28'45."W, 25.x.2010, M. Elgueta, 1 (MNNC), 18.viii.1971, L. Alfaro, 10 (MNNC), 4.ii.1975, J. Solervicens, 2 (MNNC), 18.viii.1996, F. Ramírez, 1 (MNNC). Limarí Province: Caleta Limarí, 22.ix.2004, A. Levicán, 1 (IADIZA), 1 (LEULS), 22.ix.2004, J. Pizarro-Araya, 2 (IADIZA), 15 (LEULS), 22.ix.2004, 5 (LEULS), Los Loros, Desembocadura Río Limarí, 18.ix.1969, L.E. Peña, 10 (FMNH), Socos, 22.ix.2004, J. Pizarro-Araya, 1 (IADIZA), 8 (LEULS), Punta Talca, 21xi.1967, L.E. Peña, 1 (FMNH), Quebrada El Teniente, 24–31.vii. 1960, L.E. Peña, 3 (FMNH), 26.x. 1961, L.E. Peña, 9 (FMNH), 13.i.1966, L.E. Peña, 8 (FMNH), 21.xii.1969, L.E. Peña, 4 (FMNH), 28.ix.1980, L.E. Peña, 69 (FMNH), 4 km N., Quebrada El Teniente, 15.xii.1967, L.E. Peña, 2 (FMNH), S. Puerto Manso, 18.ix.1967, L.E. Peña, 4 (FMNH), Huentelauquén, 6.ii.1969, L.E. Peña, 4 (FMNH), La Cebada, 10 m, 30°58'30.1"S, 71°38'38."W, 31.x.2010, M. Elgueta, 1 (MNNC).

### ﻿ Praocis (Mesopraocis) calderana

Taxon classificationAnimaliaColeopteraTenebrionidae

Kulzer, 1958

9C1999BB-50D3-516C-8169-B04780345E51

Figs 3A, B , 4B , 5

Praocis calderana Kulzer, 1958: 32 (rev.); Peña 1966: 431 (cat.); Vidal and Guerrero 2007: 73, 218; Pizarro-Araya et al. 2008: 273 (list.); Pizarro-Araya et al. 2012: 9 (ecol.); Flores and Pizarro-Araya 2014: 60 (list).

#### Redescription.

Length 6.25–9.00 mm. Body, antennae, legs black, dark brown to light brown, body widest at prothorax. Head. Clypeus with punctures bearing short setae on all surface, width of anterior margin equal to half the interocular width; frons with punctures bearing short setae on all surface, border of punctures not elevated; frontoclypeal suture lacking setae; eyes lacking supraocular groove; antennae reaching anterior third of lateral margin of pronotum; antennomere 9 wider than long, 11 longer than wide, antennomere 9 of equal length to 10 and shorter than 11, antennomere 9 of equal width to 10 and wider than 11, antennomere 10 wider than 11.

***Thorax*.** Pronotum with anterior angles rounded, produced, very close to eyes and epicanthus, lateral margin explanate, disc convex not reaching lateral margins (Fig. 4B); posterior margin bisinuate, central area not extending beyond lateral extents of posterior margin (Fig. 4B), posterior angles acute, pointed, produced. Hypomeron with a fringe of long golden setae below lateral margin of pronotum. Metaventrite with punctures on lateral thirds separated by one to two puncture diameters.

***Elytron*.** Pseudopleuron with sparse punctures of which emerge short, erect setae, becoming long, dense on anterior half of upper surface, punctures separated by three to six puncture diameters on lower surface, separated by one to two puncture diameters on anterior half of upper surface; epipleuron with a fringe of long golden setae on anterior half of edge, texture different than that of elytron, shiny, almost smooth, with sparse punctures and setae.

***Legs*.** Ventral surface of trochanters with single long setae. Apical process of distal margin of protibiae equal to length of protarsomeres 1–3 combined; protibial length twice the width of distal margin. Metatarsomere 1 twice as long as metatarsomeres 2–3 combined.

***Abdomen*.** Ventrites 1–3 with sparse punctures separated by two to four puncture diameters; ventrite 4 with sparse punctures separated by one to two puncture diameters; ventrite 5 with punctures on central area separated by two to four puncture diameters, on lateral thirds separated by one to two puncture diameters.

***Male genitalia* (Fig. 3A, B).** Basal lamina of tegmen proximally narrow, with base pointed. Lateral styles of tegmen with proximal margin triundulate, medially notched, with setae on distal 1/6 of ventral surface. Median lobe equal width throughout, one third of the width of tegmen, with apical aperture small.

#### Type specimens.

Holotype male: [Caldera/ (8 km SE)/ Costa Atacama/ 17.vi.1955/ Coll: L.E. Peña] [Holotypus/ *Praocis*/ *calderana* m./ det. H. Kulzer 1957] (FMNH); allotype female (FMNH) and two paratypes with the same data as holotype plus [Paratypus/ *Praocis*/ *calderana* m./ H. Kulzer 1957] (FMNH).

#### Other material examined.

Chile. Antofagasta Region: Antofagasta Province: Antofagasta. 20 km N Paposo, vi.1985, L.E. Peña, 4 (FMNH), S Paposo, sand dunes, 25°07'1.8"S, 70°28'52."W, 5.xi.2012, M. Elgueta, 3 (MNNC), 8 (IADIZA), 2 (LEULS), 17 km S Paposo, playa Las Losas, 9.iii.2017, 25.13746°S, 70.46178°W, G. Flores, R. Predel, M. Bläser, L. Ragionieri, A. Zuñiga, 18 (IADIZA), 2 (LEULS). Atacama Region: Chañaral Province: Parque Nacional Pan de Azúcar, 14.x.1992, L.E. Peña, 4.v.2003, J. Pizarro-Araya, 4 (IADIZA), 6 (LEULS), Cerro Verde, 26.29759°S, 70.64099°W, x.2017, A. Zúñiga, 2 (MNNC), 3 (IADIZA), 2 (LEULS), 20 km S Chañaral, 16.ix.2000, M. Beéche, 2 (MNNC), 15 km N Chañaral, 21.ix.2000, M. Beéche, 2 (MNNC), N Flamenco, 19 m, sand dunes, 26°32'51.9"S, 70°41'09."W, 30.x.2010, M. Elgueta, 3 (MNNC). Copiapo Province: Rodillo, 27.x.1989, H. Vásquez, 1 (IADIZA), 8 (LEULS); ix.1990, H. Vásquez, 3 (IADIZA), 11 (LEULS);15.x.1991, J. Cepeda, 2 (IADIZA), 8 (LEULS), Quebrada El León, Caldera, 1.ix.2007, J. Pizarro-Araya, 2 (IADIZA), 6 (LEULS), 01.xi.2007, P. Agusto (LEULS), Quebrada El León, Caldera, 26°57'44"S, 70°45'51"W, 21.ii.2006, J. Pizarro-Araya, 5 (LEULS), Punta Frodden, 20 km N Caldera, 26.iv.1956, L.E. Peña, 1 (FMNH), 20 km SE Caldera, 8.xii.1967, L.E. Peña, 31 (FMNH), 12 km E Caldera, 341 m, 27°06'2.8"S, 70°40'23." W, 13.xi.2010, M. Elgueta, 2 (MNNC), 10 km N Caldera, 4.x.1983, A. Roig, 4 (IADIZA), 1 (LEULS), Caldera, 17.vi.1955, L.E. Peña, 4 (MNNC), 2–4.x.1981, M. Elgueta, 6 (MNNC), Bahía Inglesa, 15.ix.2000, M. Beéche, 2 (MNNC), Bahía Inglesa, 3 m, sand dunes, 27°06'26.9"S, 70°51'03."W, 6.xi.2012, M. Elgueta, 8 (IADIZA), 2 (LEULS), Bahía Inglesa, sand dunes, 27.1°S, 70.8667°W, 24.i.2010, E. Ruiz, J. Mondaca, F.M. Alfaro (3 IADIZA), Boca Río Copiapó, 13.vi.1968, L.E. Peña, 1 (FMNH), 40 km S Copiapó, 25.x. 1983, L.E. Peña, 1 (FMNH), Puerto Viejo, 10.x.1980, L.E. Peña, 3 (FMNH), ix.1982, L.E. Peña, 9 (FMNH), Ruta 5 norte con Puerto Viejo, 157 m, 27°21'34"S, 70°40'"W, 24.i.2010, E. Ruiz Manzanos, F.M. Alfaro, J. Mondaca, 1 (IADIZA). Huasco Province: Playa Blanca, 11.iii.2017, 26.17352°S, 70. 66057°W, G. Flores, 1 (IADIZA), Carrizal Bajo, Vallenar, xi.1991, H. Vásquez, 8 (LEULS), Parque Nacional Llanos de Challe, 15.x.1997, pitfall traps 1 (LEULS). Coquimbo Region: Elqui Province: Chungungo, Playa Blanca, 27.ii.2005, J. Pizarro-Araya, 3 (LEULS), Lagunillas, Coquimbo, xi.1990, pitfall traps 11 (LEULS).

### ﻿ Praocis (Mesopraocis) nitens

Taxon classificationAnimaliaColeopteraTenebrionidae

Kulzer, 1959

11ECA462-290D-5362-BC20-BAE4DB0E3ADE

Figs 3C, D , 4C , 5

Praocis nitens Kulzer, 1959: 561; Peña 1966: 431 (cat.); Vidal and Guerrero 2007: 73, 218; Pizarro-Araya et al. 2008: 273 (list.); Flores and Pizarro-Araya 2014: 60 (list).

#### Redescription.

Length 7.87–10.62 mm. Body, antennae, legs black to dark brown, body widest at prothorax. Head. Clypeus with punctures bearing short setae on all surface, width of anterior margin equal to half the interocular width; frons with punctures bearing short setae on all surface, border of punctures on frons and epicanthus elevated; frontoclypeal suture with short or long setae; eyes with supraocular groove; antennae reaching anterior third of lateral margin of pronotum; antennomere 9 longer than wide, 11 wider than long, antennomere 9 longer than 10 and 11, antennomere 9 narrower than 10 and wider than 11, antennomere 10 wider than 11.

***Thorax*.** Pronotum with anterior angles rounded, not produced, remote from eyes and epicanthus, lateral margins declivous, disc convex reaching lateral margins (Fig. 4C); posterior margin bisinuate, central area not extending beyond lateral extents of posterior margin (Fig. 4C), posterior angles right with apex rounded, produced. Hypomeron with a fringe of long golden setae below lateral margin of pronotum. Metaventrite with punctures on lateral thirds separated by two to four puncture diameters, transverse grooves parallel to metacoxae formed by the fusion of a row of punctures.

***Elytron*.** Pseudopleuron with sparse punctures of which emerge long, erect setae, becoming long, dense on anterior half of upper surface, punctures separated by three to six puncture diameters on lower surface, separated by one to two puncture diameters on anterior half of upper surface; epipleuron with a fringe of long golden setae on anterior half of edge, shiny, almost smooth, with sparse punctures and setae on anterior quarter, and with a row of punctures with same diameter and depth as on pseudopleuron located on posterior three fourths.

***Legs*.** Ventral surface of protrochanters with hair brush. Apical process of distal margin of protibiae equal to length of protarsomeres 1–2 combined; protibial length 2.5 times the width of distal margin. First metatarsomere 1.4–1.6 times longer than metatarsomeres 2–3 combined (Fig. 4C).

***Abdomen*.** Ventrites 1–2 with sparse punctures separated by two to four puncture diameters; ventrites 3–4 smooth on central area, with punctures on lateral thirds separated by two to four puncture diameters; ventrite 5 with punctures on central area separated by two to four puncture diameters, on lateral thirds separated by one to two puncture diameters.

***Male genitalia* (Fig. 3C, D).** Basal lamina of tegmen equal width throughout, with base concave. Lateral styles of tegmen with proximal margin bisinuate, with setae on distal 1/6 of ventral surface. Median lobe distally widened, half the width of tegmen, with apical aperture large.

**Figure 5.**
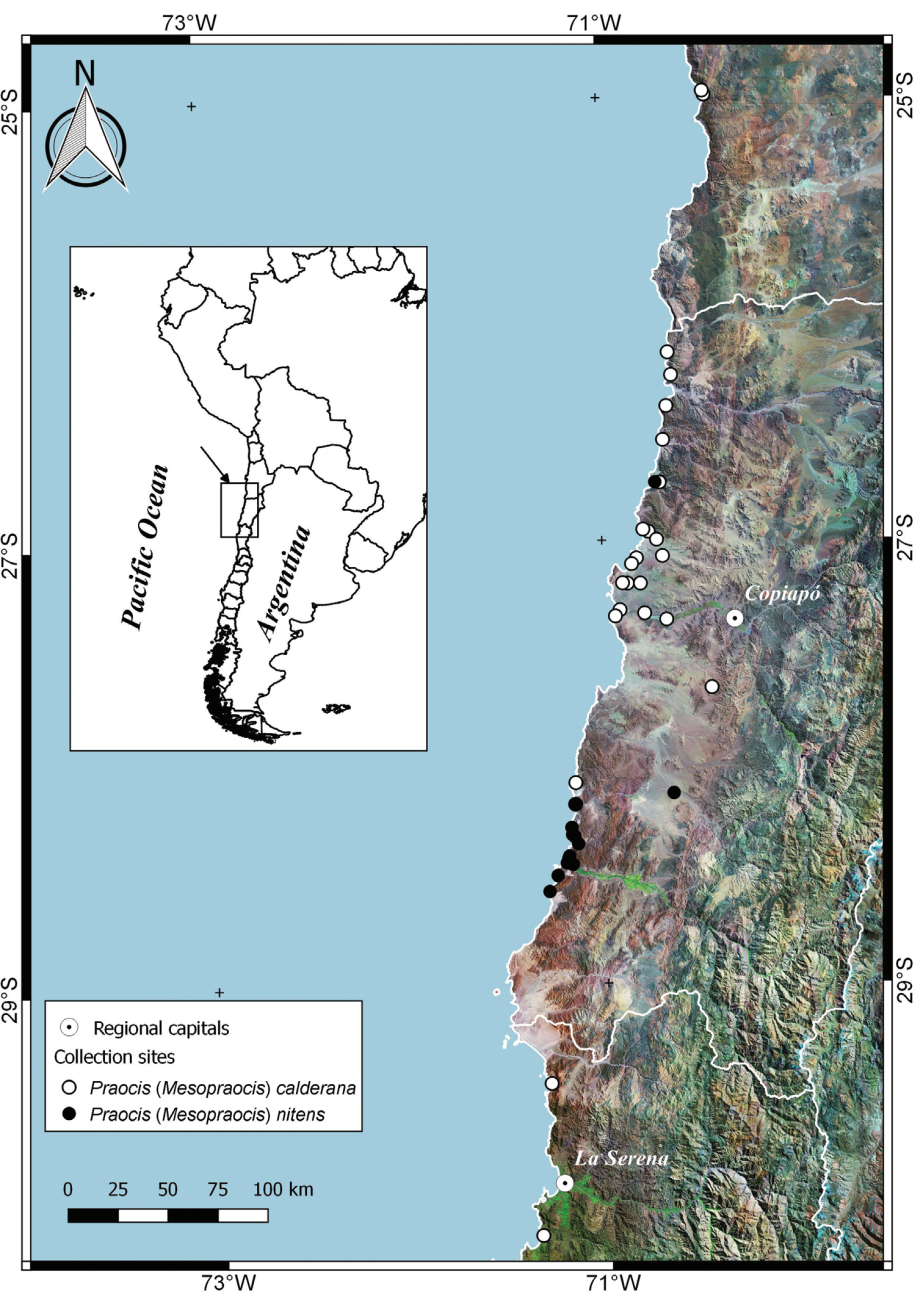
Geographical distribution of Praocis (Mesopraocis) calderana and Praocis (Mesopraocis) nitens.

#### Type specimens.

Holotype male: [Huasco/ Atacama/ 20–22.x.1957/ Coll: L.E. Peña] [Holotypus/ *Praocis*/ (*Mesopraocis*)/ *nitens* m./ det. H. Kulzer 1958] (FMNH); allotype female (FMNH) and four paratypes with the same data as holotype plus [Paratypus/ *Praocis*/ *nitens* m./ H. Kulzer 1958] 2 (FMNH), 2 (OSUC).

#### Other material examined.

Chile. Atacama Region: Copiapo Province: Rodillo, Caldera, xi.1991, H. Vásquez, 10 (LEULS). Huasco Province: Algarrobal, Atacama, 15.ix.2002, J. Pizarro-Araya, 1 (LEULS), Quebrada Carrizalillo, N. Huasco, 12.x.1980, L.E. Peña, 1 (FMNH), Parque Nacional Llanos de Challe, 28°11'17.36"S, 71°9'13.63"W, 20–22.xii.2016, J. Pizarro-Araya & F.M. Alfaro, 9 (LEULS), 15.x.1997, Traps barber, 17 (LEULS), Parque Nacional Llanos de Challe, UTM 19 J 311128 W, 6883333 N, 269 msl, 31.viii-03.ix.2017, J. Pizarro-Araya & F.M. Alfaro (SIMEF-Project), 3 (LEULS), Agua de Luna, 20 km N Huasco, sand dunes, 28°20'09"S,71°09'"W, i.2004, F.M. Alfaro, 1 (IADIZA), 27.x.2010, M. Elgueta, 4 (MNNC), 28°20'5.2"S, 71°09'37."W, 29.x.2012, M. Elgueta, 2 (IADIZA), Huasco bajo, 8.viii.1976, 1 (UCCC), Huasco, 20–22.x.1957, L.E. Peña, 2 (OSUC), 29 (FMNH). **Note**: these 29 specimens were later labeled as paratypes by Peña but these were not originally designated by Kulzer (1959), who stated that he designated 24 paratypes with the same data as holotype and Peña kept 88 more specimens; 17–21.x.1974, L.E. Peña, 68 (FMNH), Huasco, sand dunes, 28–29.ix.1981, M. Elgueta, 3 (MNNC), 28°27'19.5"S, 71°12'6."W, 28.x.2012, M. Elgueta, 1 (IADIZA), 5 km N Huasco, 13.ix.1996, Leg. G. Castillo, 1 (LEULS), Agua de Luna, Huasco, 12–16.xi.2008, Leg. F. M. Alfaro, 5 (LEULS), i.2004, F.M. Alfaro 4 (LEULS), Caleta Angosta, Huasco, 18.viii.2009, F. M. Alfaro, 1 (LEULS), Tres Playitas, Huasco, xi.2015, F.M. Alfaro, 1 (IADIZA), 16–21.xi.2014, 28°25' 26.1"S, 71°11'19.5"W, J. Pizarro-Araya & F.M. Alfaro, 10 (LEULS), Los Bronces, Huasco, 15.x.2006, F.M. Alfaro, 3 (IADIZA), 15.x.2006, 6 (LEULS), Playa Tontado, Huasco, 28.xi.2008, Leg. F.M. Alfaro, 5 (LEULS).

### ﻿ Praocis (Mesopraocis) arenicola sp. nov.

Taxon classificationAnimaliaColeopteraTenebrionidae

AA10F93A-EDA3-5464-AEDE-D5AA0B500BF8

http://zoobank.org/A4443F35-5686-4693-8F04-D35694CD841C

Figs 4D , 6

#### Etymology.

The specific epithet is a Latin adjective, referring to the fact that this species inhabits sandy places.

#### Diagnosis.

Pronotum with central area of posterior margin not extending beyond lateral extents of posterior angles; body widest at prothorax; width of anterior margin of clypeus equal to half the interocular width. Praocis (Mesopraocis) arenicola sp. nov. superficially resembles Praocis (Mesopraocis) nitens by having pronotum with lateral margins declivous, disc convex reaching lateral margins; eyes with supraocular groove; antennomere 9 longer than wide or equal length than wide, and longer than 10. Praocis (Mesopraocis) arenicola sp. nov. differs from P. (M.) nitens by having frons, epicanthus, elytron with shallow punctures; frons and epicanthus with puncture depth smaller than diameter; elytron with puncture depth the same size than diameter; antennomere 9 of equal length than 11 and of equal width to 10; antennomere 11 longer than wide. Praocis (Mesopraocis) nitens has frons, epicanthus, elytron with impressed punctures; frons and epicanthus with puncture depth the same size than diameter; elytron with puncture depth larger than diameter; antennomere 9 longer than 11, narrower than 10; antennomere 11 wider than long.

#### Description.

Length 6.62–10.00 mm. Body, antennae, legs black to dark brown, body widest at prothorax. Head. Clypeus with punctures bearing short setae on all surface, width of anterior margin equal to half the interocular width; frons with punctures bearing short setae on all surface, border of punctures not elevated; frontoclypeal suture with short setae; eyes with supraocular groove; antennae reaching anterior third of lateral margin of pronotum; antennomeres 9 and 11 longer than wide, antennomere 9 longer than 10 and shorter than 11, antennomere 9 of equal width to 10 and wider than 11, antennomere 10 wider than 11.

***Thorax*.** Pronotum with anterior angles rounded, not produced, remote from eyes and epicanthus, lateral margins explanate, disc convex not reaching lateral margins (Fig. 4D); posterior margin bisinuate, central area not extending beyond lateral extents of posterior margin (Fig. 4D), posterior angles right or acute with apex rounded, produced. Hypomeron with a fringe of long golden setae below lateral margin of pronotum. Metaventrite with punctures on lateral thirds separated by one to two puncture diameters.

**Figure 6.**
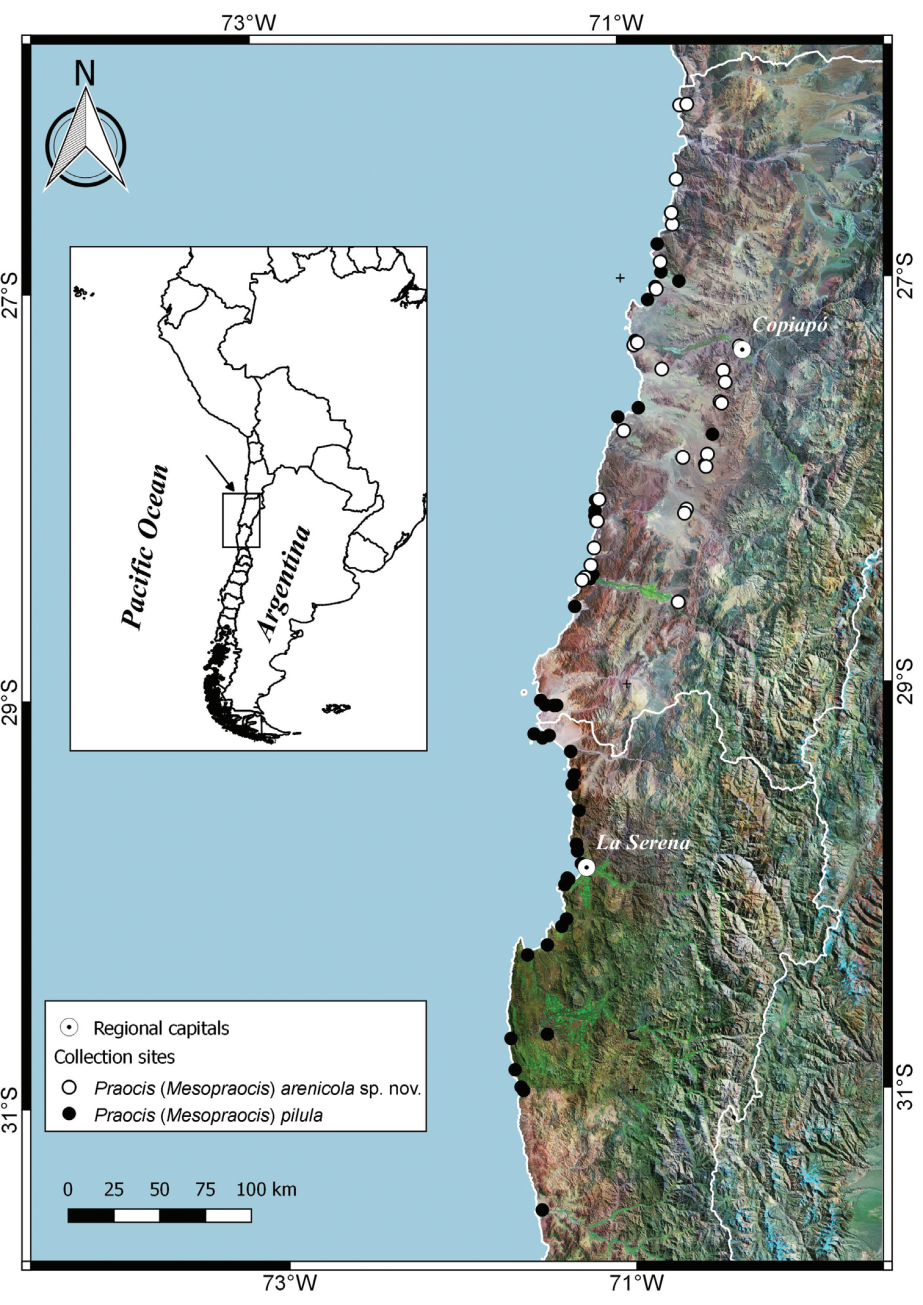
Geographical distribution of Praocis (Mesopraocis) pilula and Praocis (Mesopraocis) arenicola sp. nov.

***Elytron*.** Pseudopleuron with sparse punctures from which emerge long, erect setae, becoming a dense fringe on anterior half of upper surface, punctures separated by three to six puncture diameters on lower surface, separated by one to two puncture diameters on anterior half of upper surface; epipleuron with a fringe of long golden setae on anterior half of edge, almost smooth, with sparse punctures and setae on anterior quarter and with a row of punctures smaller diameter and depth than on pseudopleuron located on posterior three fourths.

***Legs*.** Ventral surface of protrochanters with hair brush. Apical process of distal margin of protibiae equal to length of protarsomeres 1–3 combined; protibial length 2.5 times the width of distal margin. First metatarsomere 1.4–1.6 times longer than metatarsomeres 2–3 combined.

***Abdomen*.** Ventrites 1–3 with sparse punctures separated by two to four puncture diameters; ventrite 4 with sparse punctures separated by one to two puncture diameters; ventrite 5 with punctures on central area separated by two to four puncture diameters, on lateral thirds separated by one to two puncture diameters.

**Male genitalia.** Basal lamina of tegmen equal width throughout, with base concave. Lateral styles of tegmen with proximal margin concave, with setae on distal 1/6 of ventral surface. Median lobe equal width throughout, one third the width of tegmen, with apical aperture small.

#### Type specimens.

Holotype male: [Chile: III Región, Chañaral/ sur Flamenco dunas/ 58m 26°35'55.9"S, 70°35'45"W, 30.x.2012/ Coll: Mario Elgueta] [Praocis (Mesopraocis)/ *arenicola* sp. nov./ HOLOTYPUS male/ Det. G. Flores and/ J. Pizarro-Araya 2021] (MNNC); allotype female (MNNC) and one paratype male with the same data as holotype (IADIZA). Paratypes. CHILE, Atacama Region: Chañaral Province: Parque Nacional Pan de Azúcar, 4.v.2003, J. Pizarro-Araya, 1 (LEULS), Los Sapos km 729, 28°01'42.2"S, 70°33'01."W, 532 m 8.xi.2003, C. Mattoni, L. Prendini, J. Ochoa, 1 (IADIZA), Rodillo, Caldera, ix.1991, pitfall traps, 2 (LEULS), 1 (MNNC), Flamenco, Caldera, 3.v.2003, J. Pizarro-Araya, 2 (LEULS). Copiapo Province: Punta Frodden, 20 km N. Caldera, Atacama, 26.iv.1956, L.E. Peña, 2 (FMNH), Punta Frodden, Atacama, 2.iv.1974, L.E. Peña, 3 (FMNH), Caldera, Atacama, 17.vi.1955, L.E. Peña, 1 (FMNH), Copiapó, Fundo Santa Elena, 19–23.vii.1982, E. Arredondo, 3 (UCCC), Bahía Inglesa sand dunes 3 m, 27°06'27.2"S, 70°51'3."W, 6.xii.2012, M. Elgueta, 2 (IADIZA), 3 (MNNC), Copiapó 15 km S, 15.vii.1952, L.E. Peña, 2 (FMNH), Copiapó, 5.x.1968, L.E. Peña, 3 (FMNH), Copiapó 80 km S, 5.x.1966, L.E. Peña, 10 (FMNH), 1 (NHMB), 1 (HNHM), 40/60 km S Copiapó, 25.vi.1968, L.E. Peña, 9 (FMNH), 30 km S. Copiapó, 31.i.1967, G. Monsalve, 7 (FMNH), S. Copiapó, 20.vi.1968, P. Vidal, 1 (FMNH), 100 km S. Copiapó Atacama, 23.x.1983, L.E. Peña, 5 (FMNH), Copiapó S. km 700, Atacama, 1.i.1980, L.E. Peña, 1 (FMNH), Boca Río Copiapó, 13.vi.1968, L.E. Peña, 1 (FMNH), Torres del Inca, Copiapó, 26.60759°S, 70.69839°W, x.2017, A. Zúñiga 1 (MNNC), 1 (IADIZA), N. Estación Castilla, 25.xi.1972, L.E. Peña, 1 (FMNH), Quebrada El Morel, x-xi.2009, J. Pizarro-Araya & F.M. Alfaro, 2 (LEULS), 2 (MNNC), 1 (UCCC), Huasco Province: Vallenar a Copiapó, 18.ix.1963, L.E. Peña, 1 (FMNH), Algarrobal, Atacama,15.ix.2002, J. Pizarro-Araya 1 (LEULS), Huasco, Atacama, 12.xii.1967, L.E. Peña, 4 (FMNH), Quebrada Maitencillo, Vallenar, 11.x.1980, L.E. Peña, 1 (FMNH), Puerto Viejo, Atacama, 20.ix.1982, L.E. Peña 1 (FMNH), Puerto Viejo, Atacama, 10.x.1980, L.E. Peña, 1 (FMNH), Algarrobal, 3.xii.2002, J. Pizarro-Araya, 1 (IADIZA), 15.ix.2002, J. Pizarro-Araya, 1 (LEULS), Carrizal Bajo, 9–29.ix.1989, Traps Barber, 1 (IADIZA), Carrizal Bajo, 29.xi-29.x.1989, Traps Barber, 1 (IADIZA), Carrizal Bajo, 29.x.1989, H. Vásquez, 1 (IADIZA), Carrizal Bajo, 29.x.1989, H. Vásquez, 1 (IADIZA), Carrizal Bajo, ix.1991 bajo/ arbusto, H. Vasquez, 4 (IADIZA), 1 (LEULS), Carrizal Bajo, Huasco, ix.1991, H. Vásquez, 3 (IADIZA), Llanos de Challe/ Vallenar/ 15.x.1997 pitfall traps, 3 (IADIZA), Parque Nacional Llanos de Challe, dunas interiores, 18.x.2014, J. Pizarro-Araya, 3 (LEULS), 3 (MNNC), 3 (UCCC), Parque Nacional Llanos de Challe, 19 J 0294745, 6880089, 221 msl, 31.viii-03.ix.2017, J. Pizarro-Araya & F.M. Alfaro (SIMEF-Project), 2 (LEULS), Aguada de Tongoy, Huasco, 19.xi.2005, F.M. Alfaro, 1 (LEULS), Tres Playitas, Huasco, xi.2015, F.M. Alfaro, 1 (LEULS), El Pimiento, Vallenar, 2.vi.1968, L.E. Peña, 1 (FMNH), Guacolda, Huasco, xi.2015, J. Pizarro-Araya & F.M. Alfaro, 2 (LEULS), 3 (MNNC), 2 (UCCC).

#### Other material examined.

Chile. Atacama Region: Copiapo Province: Torres del Inca, 26.60759°S, 70.69839°W, x.2017, Coll: A. Zúñiga, 2 (IADIZA).

## ﻿Morphological adaptations to fossorial life and comparison with other genera of psammophilous Tenebrionidae

Members of Praocis (Mesopraocis) inhabit loose sands of coastal grassy dunes covered with more or less scattered vegetation and according to the Koch’s (1961) classification of psammophilous species, they are plant-followers. In this microhabitat, the active vegetation with its favourable shade conditions and high-water content influences the mixture of sand and detritus between the roots (Koch 1961).

All species of Praocis (Mesopraocis) spend most of their lives within the substrate (larvae, pupae, and adults) and they possess many of the morphological modifications described by Koch (1961) and Doyen (1984) for “sand swimming” tenebrionids. All these characters in adults represent adaptations to fossorial life in weakly consolidated sands or dunes stabilised with poor vegetation (Matthews et al. 2010). The most striking modifications are in body shape, setation, structure of legs, and antennae.

Body subglobular. This shape of the body increases the volume of the subelytral cavity which is hypothesised to be a protection from heat and to reduce respiratory water loss (Cloudsley-Thompson 2001). The air used for respiration is believed to pass first through the subelytral cavity and it is thus possibly cooled and moistened before entering the spiracles (Duncan 2003). The subglobose shape of the elytra in Praocis (Mesopraocis) species (Fig. 4A–D) places the pseudopleuron in horizontal position (Fig. 1F), conditioning to horizontal position the middle and hind legs for gliding movements (Koch 1961). This was also observed in fossorial species of *Eusattus* LeConte by Doyen (1984) and *Trachyscelis* Latreille (Nabozhenko and Purchart 2017). In comparison, epigean species of *Eusattus* (Doyen 1984; collection specimens IADIZA) and Praocis (Praocis) (Flores and Pizarro-Araya 2012) have the pseudopleuron in oblique position, which allows middle and hind legs’ cursorial movements.

Very short antennae. In taxa that burrow through loose sand, the antennae often become shortened, sometimes with the fusion of segments (Doyen 1984) decreasing the usual number of eleven (Matthews et al. 2010). Shortened antennae are present in all species of Praocis (Mesopraocis) reaching only to the anterior third or quarter of the lateral margin of the pronotum (Fig. 4A–D). In addition, specimens of Praocis (Mesopraocis) pilula can exhibit a different number of antennomeres (9, 10 or 11), result of the fusion or lack of intermediate segments (Fig. 1B–D). Similarly, fossorial species of *Trachyscelis* also have short antennae 10-segmented (Nabozhenko and Purchart 2017) and the most highly modified species of *Eusattus* to fossorial life present shortening of the antennae (Doyen 1984). In contrast, epigean species of *Eusattus* (Doyen 1984; collection specimens IADIZA) and Praocis (Praocis) (Flores and Pizarro-Araya 2012) have longer antennae, extending midpoint to 3/4 length of lateral margin of pronotum.

Foretibia shape. In sand-burrowing or sand-swimming forms, the foretibiae are commonly specialized as lamellate digging tools as in *Eusattusmuricatus* and *ciliatus* species groups (Doyen 1984). The foretibiae in all species of Praocis (Mesopraocis) are explanate, gradually expanded apically as an attenuate process, concave from behind (Fig. 2A, B), with hypertrophic development of the tactile armatures in outer and apical margins which are in continuous contact with the loose grains of sand (Koch 1961). Foretibiae of Praocis (Mesopraocis) species have two types of conical pegs mentioned in generic diagnosis (Fig. 2C, D). Foretibiae of *Trachyscelis* species also exhibit a protibial lamina explanate with two types of conical pegs (Nabozhenko and Purchart 2017) named spines by the authors. In comparison, ambulatory species of *Eusattus* and Praocis (Praocis) present the foretibiae slightly flattened apically (Doyen 1984; Flores and Pizarro-Araya 2012).

Pronotal setal fringe. Burrowing Pimeliinae often have fimbriate lateral body margins. Usually the setae are set on the lateral margin of pronotum (= carina of Doyen 1984: Fig. 25), but in all species of Praocis (Mesopraocis) setae are inserted on the hypomeron well below the lateral margin of pronotum, forming a prominent pronotal fringe (Fig. 1E). Elytral pseudopleuron in all Praocis (Mesopraocis) species is fringed by a tuft of long golden setae on upper surface (Fig. 1F). Both pronotal and elytral rows of long golden setae form a continuous setaceous fringe surrounding the body, which assist forward movement through the substrate. These features are also observed in fossorial species of *Eusattus* (Doyen 1984) and *Trachyscelis* (Nabozhenko and Purchart 2017). On the other hand, epigean species of *Eusattus* (*reticultatus* and *convexus* species groups) and Praocis (Praocis) have the hypomeron and elytral pseudopleuron glabrous except for submarginal fringe of short setae (Doyen 1984; Flores and Pizarro-Araya 2012).

All species of Praocis (Mesopraocis) have elytral surface texture smooth, lacking rugosity and tubercles, devoid of carinae or striae, and lateral margin not defined (Fig. 4A–D); these features are important in fossorial forms in reducing friction against the substrate (Doyen 1984), characteristic also of fossorial species of *Eusattus* and *Trachyscelis* (Doyen 1984; Nabozhenko and Purchart 2017). The surface dwellers species of *Eusattus* (*reticultatus* and *convexus* species groups) and Praocis (Praocis) present the elytra carinate, sulcate, punctato-rugulose to coarsely rugose or scabrous and lateral margin well defined (Doyen 1984; Flores and Pizarro-Araya 2012).

It has been hypothesised that psammophily has evolved independently in a number of unrelated taxa (Matthews et al. 2010). In this study we have compared the characters of adaptation to psammophilic life of Praocis (Mesopraocis) species with other phylogenetically unrelated groups that have similar lifestyles, whose morphology is well known (Doyen 1984; Nabozhenko and Purchart 2017) and inhabit similar environments in areas of sandy substrate. It is possible to conclude that these groups, due to external environmental pressures, have developed the same adaptations for life in the sand. It is also the first time that the characters of psammophilous species are described, analysed and illustrated in South American tenebrionids.

## Supplementary Material

XML Treatment for Praocis (Mesopraocis)

XML Treatment for Praocis (Mesopraocis) pilula

XML Treatment for Praocis (Mesopraocis) calderana

XML Treatment for Praocis (Mesopraocis) nitens

XML Treatment for Praocis (Mesopraocis) arenicola
